# Introduction of a Less Invasive Revision Rhinoplasty Using Closed Nasal Chondrotome

**DOI:** 10.29252/wjps.8.1.108

**Published:** 2019-01

**Authors:** Amir Manafi, Zahra Sadat Hamedi, Farzad Manfai

**Affiliations:** School of Medicine, Iran University of Medical Sciences, Tehran, Iran;

**Keywords:** Closed nasal chondrotome, Revision rhinoplasty, Dorsal spur

## Abstract

Rhinoplasty is the most common aesthetic surgical procedure in Iran. Dorsal bony and cartilaginous structures of the nose play an important role in nasal esthetics and function. Manipulation of nasal dorsum is one of the cardinal procedures during rhinoplasty operation. Some cases of primary rhinoplasty lead to various post-operative nasal imperfections. One of the most common imperfections is dorsal nasal spur which can cause dorsal irregularities. The accurate rate of imperfections relates to some factors like the surgeon’s experience and his/her precision, and appropriate post-operative patient care. Alomost 15%of revision or secondary surgical manipulations are acceptable for an experienced plastic surgeon. Most of the revision rhinoplasties are due to minor deformities in nasal dorsum like cartilaginous spur or mild focal depression. We have introduced an innovative device “Closed Nasal Chondrotome” that can ease the procedure for treating of minor nasal dorsal deformities. we propose the use of closed nasal chondrotome for mild dorsal spurs and have presented the effectiveness of this device in one patient. This simple but very effective instrument can be an alternative for a revision rhinoplasty procedure in the operating room to an outpatient procedure with local anesthesia. This method has been used in one patient with the satisfactory result, permitting corrections of minor cartilaginous excess deformities, with a less invasive procedure.

## INTRODUCTION

Management of the cartilaginous structures of the nose has always been a challenge for the plastic surgeon. Most often, over long-term follow-up of the rhinoplasties, the sharp edges and irregularities of the cartilaginous handling become visible, especially in patients with thin skin. As the rhinoplasties have evolved, more concern has been paid to the important role of these structures both in the aesthetics and function of the nose. To achieve a better conformation of the dorsum nasal cartilages were treated by partial resections since the beginning of aesthetic rhinoplasties.^1^ As the rhinoplasties became more conservative in the 1990s, the conforming stitches of the alar cartilages were introduced, helping to achieve a better contour without ample resections or sharp edge grafts.^2^

Revision and secondary rhinoplasty is probably one of the most difficult procedures performed in facial plastic surgery. Many of these postsurgical deformities are found in the nasal dorsum. These changes are usually the result of inadequate or overzealous resections as well as inappropriate healing. Most of these patients are operated on through the open approach, and treatment is multifactorial. Osteotomy and rasp techniques are used to realign; cartilage grafts are used to fill in, camouflage, and smooth out; and in severe cases implants are used to fill in large defects.^3^ Our experience with the use of the “Closed Nasal Chonrotome” for reshaping the dorsal nasal cartilages is presented. This device allows a very controllable, altering and recorrecting of the shape and irregularities of dorsal nasal cartilage an easy manner.

## CASE REPORT

**Fig 1:**
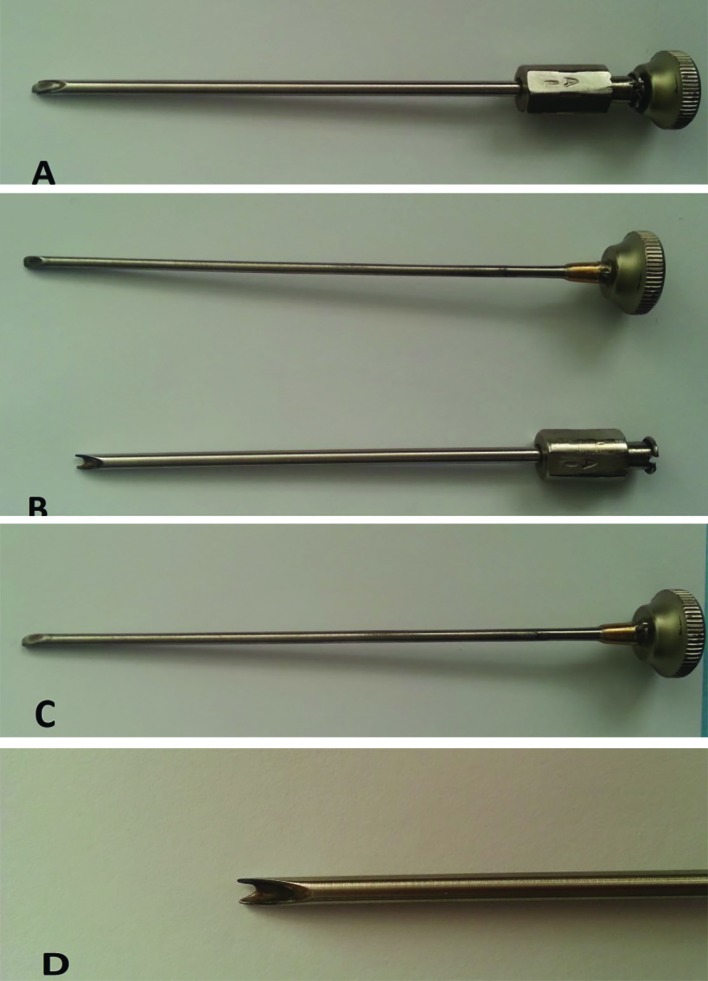
A) Closed Nasal Chondrotome, 12 cm length and 5 mm diameter. B) The instrument consisted of two part: sheet and the obturator. C) Obturator tool with an oblique and sharp head for removing the spurs. D) Head of the sheet (Close up view), with a semicircle surface which lodged the spur

**Fig 2:**
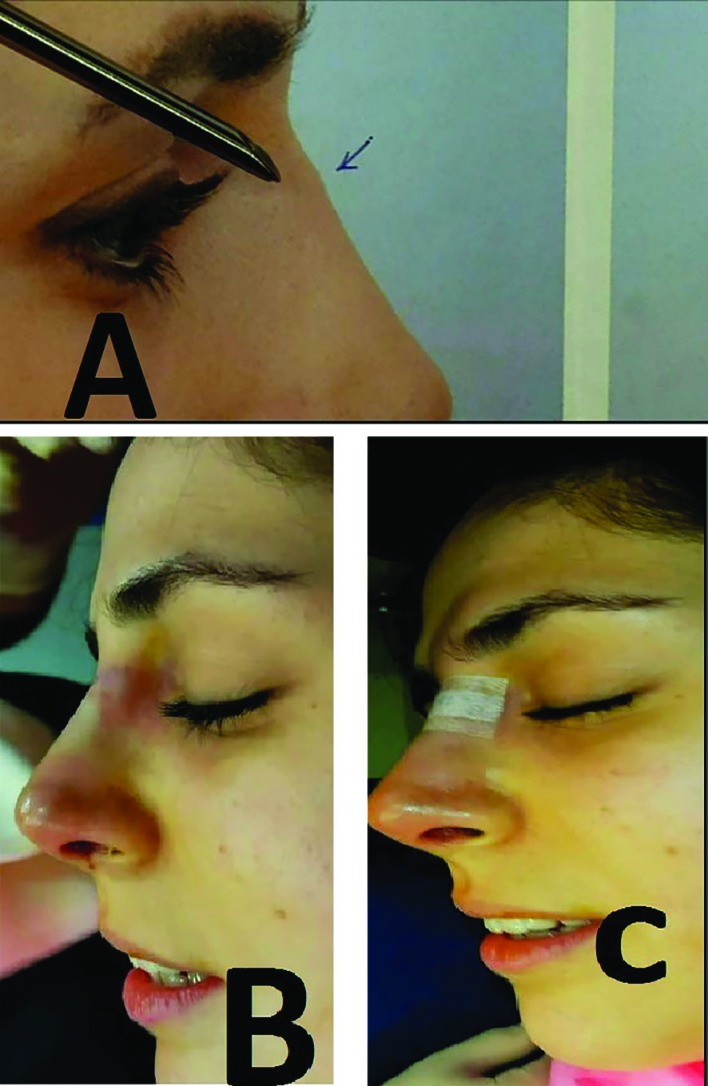
Cartilage spur in the dorsum the nose that was removed by a chondrotome. A) Before the procedure. B) Just after spur removal

A 27 years old female was treated to correct a cartilaginous irregularity of nasal dorsum. The previous surgery was done at 1 year before this procedure and the same surgeon of primary rhinoplasty has used this new device for correction of the irregularities. The cartilage resecting was done with a handy device which was named by us as “closed Nasal Chondrotome” (Figure 1). Local anesthesia was done by injection of xylocaine topical solution into the nose. The patient was alert during the whole procedure with no pain and no discomfort. The surgeon began to push the device toward the cartilaginous spur via an endonasal approach and lodge the semicircle edge of the instrument to the spur and tried to cut the bulge spur by introducing the obturator of the device across the dorsum of the nose. The surgeon could assess the shape of cartilage and the integrity of that via the touching of the dorsum by his/her second hand. This procedure took 10 minutes to be completely undertaken. The patient achieved satisfactory results (Figure 2). 

## DISCUSSION

The nasal cartilage plays an important role in nasal function and esthetics. Plastic surgeons are increasingly seeking to preserve as much as possible of both the septal and the alar cartilages that present themselves in multitude shapes and sizes.^4^^,^^5^ There is a multitude of shapes and sizes of the nasal cartilage especially on the lateral crura, and even in the same individual, the alar cartilages may be very different from each other.^1^^,^^5^

According to Lee *et al.*’s findings, the most common nasal imperfections are seen after primary rhinoplasty examination was dorsal asymmetry in 65 percent of the patients, wide dorsum in 47 percent, nostril asymmetry in 41 percent, wide alar base in 38 percent, dorsal hump in 30 percent, septal deviation in 29 percent, wide nostril in 28 percent, alar base asymmetry in 27 percent, columella protrusion in 25 percent, and wide lower lateral cartilages in 24 percent of the patients.^6^ The single most difficult challenge in revision rhinoplasty is the psychologic status of the patient.^7^

The reconstruction of the dorsum of the nose is a particular challenge given the risk of asymmetry, irregularities in contour. Even subtle irregularities in any of these features may be noticeable to the patient and to others.^8^ In our patient, dorsal nasal reconstruction after surgery resulted in a consequent nasal dorsal bump. On the long-term follow-up after primary rhinoplasty, the skin coverage tends to get thinner, and consequently, all the irregularities underneath may become visible.^9^ Injectable filler agents can sometimes be used to correct small defects in contour on the nose.^10^^,^^11^ However, the potential risk of a vascular compromise due to placing a filler product either directly into a vessel (leading to occlusion) or placing the filler product in sufficient quantity around a vessel causing compression is still a concern.^12^

The use of closed nasal chondrotome for treating the focal protrusions of nasal dorsal cartilages adds a new instrument of rhinoplastic operation armamentarium. Plastic surgeons can use this new device to remove the spur easily and avoid a second surgery. The controlled resection of the nasal cartilages with this instrument enhances or smoothes natural curvatures, without the need for a major surgery. It allows the surgeon to remove the extra cartilage that causes the irregularity. Preservation of the integrity of the nasal cartilages are very important as they play important roles in nasal esthetics and function and the surgeons can utilize it as an alternative for secondary surgery and to satisfy their patient. It reduces the cost of surgical manipulation for the patient, surgeon, and the hospitals. It helps to leave operating room free for other emergency and elective surgeries. The device also can decrease the rate of hospitalization, reduces level of anxiety for patients and their families, saving time for the patient and the surgeon.

## CONFLICT OF INTEREST

The authors declare no conflict of interest.
